# Oral vaccination of dogs: a well-studied and undervalued tool for achieving human and dog rabies elimination

**DOI:** 10.1186/s13567-018-0554-6

**Published:** 2018-07-13

**Authors:** Florence Cliquet, Anne-Laure Guiot, Michel Aubert, Emmanuelle Robardet, Charles E. Rupprecht, François-Xavier Meslin

**Affiliations:** 1 French Agency for Food, Environmental and Occupational Health & Safety (ANSES), Nancy Laboratory for Rabies and Wildlife, European Union Reference Laboratory for Rabies, European Union Reference Laboratory for Rabies Serology, OIE Reference Laboratory for Rabies, WHO Collaborating Centre for Research and Management in Zoonoses Control, Technopôle agricole et vétérinaire de Pixérécourt, CS 40009, 54220 Malzéville, France; 2 Conseils en Pharmacie et Biologie, 2 place des Quatre Vierges, 69110 Sainte Foy les Lyon, France; 3 1088 chemin des Maures, 83440 Callian, France; 4 LYSSA LLC, Lawrenceville, GA USA; 5 Neglected Zoonotic Diseases, World Health Organization, Geneva, Switzerland

## Abstract

The mass vaccination of dogs is a proven tool for rabies prevention. Besides parenteral delivery of inactivated vaccines, over the past several decades, several self-replicating biologics, including modified-live, attenuated and recombinant viruses, have been evaluated for the oral vaccination of dogs against rabies. Vaccines are included within an attractive bait for oral consumption by free-ranging dogs. Due to the high affinity between dogs and humans, such biologics intended for oral vaccination of dogs (OVD) need to be efficacious as well as safe. Baits should be preferentially attractive to dogs and not to non-target species. Although many different types have been evaluated successfully, no universal bait has been identified to date. Moreover, high bait acceptance does not necessarily mean that vaccine efficacy and programmatic success is predictable. The use of OVD in the laboratory and field has demonstrated the safety and utility of this technology. Within a One Health context, OVD should be considered as part of a holistic plan for the global elimination of canine rabies.

**Electronic supplementary material:**

The online version of this article (10.1186/s13567-018-0554-6) contains supplementary material, which is available to authorized users.

## Introduction

More than a century after Louis Pasteur developed a rabies vaccine, the disease remains endemic worldwide. This acute progressive encephalitis causes approximately 60 000 (95% confidence intervals 25–159 000) human fatalities annually—approximately one death every 10 min—and over 3.7 million (95% CIs 1.6–10.4 million) disability-adjusted life years (which incorporates both premature mortality and disability). The vast majority of these estimated human rabies cases occurs in Africa (36.4%) and Asia (59.6%) [1]. More than 99% of all rabies human cases are transmitted by dog bites. Forty percent of people bitten by suspect rabid animals are children younger than 15 years of age [2].

Parenteral mass dog vaccination programs associated with strict prophylactic measures have been effective in eliminating rabies in dogs in all developed countries, but prevention, control and eventually elimination of canine rabies has not been achieved in most developing countries. Low priority due to a lack of awareness of the rabies burden, epidemiological constraints, inaccessibility of some subpopulations of dogs to parenteral vaccination using injectable inactivated vaccines and limited resources were identified as some of the main obstacles for effective canine rabies prevention and control [3].

Rabies prevention by oral vaccination of wildlife with live vaccines has proven a powerful tool to eliminate or control rabies in multiple countries in Europe and North America [4, 5].

This approach has been proposed as a complementary policy to parenteral vaccination of dogs to increase overall vaccination coverage, especially in areas having large populations of non-accessible animals. However, vaccines need to be carefully selected and distribution methods require adaptation to make the technique both safe and efficacious for dogs.

Since 1988, the World Health Organisation (WHO) has supported numerous expert consultations regarding research coordination on oral vaccination of dogs (OVD) with the objective to promote the development and use of safe and effective rabies vaccines and baits. Guidelines were issued for the evaluation of candidate vaccines for efficacy and safety as well as for the development of vaccine baits [6–14]. In addition, guidelines were elaborated for the standardization of protocols to evaluate baiting systems and baiting strategies, for the organization of field trials, and for the study of dog ecology. Recently, the World Organisation for Animal Health (OIE) has shown a renewed interest for OVD [15]. The OIE now endorses the concept of OVD, which is now included in the rabies chapter of the OIE Manual of Diagnostic Tests and Vaccines for Terrestrial Animals [16]. Additionally, the Partners for Rabies Prevention provided a Blueprint for Rabies Prevention and Control [17], giving recommendations on various aspects of preventing human deaths and controlling animal rabies, including OVD [18].

The objective of this manuscript is to review the main studies conducted to evaluate different vaccine candidates for OVD according to the criteria established by international organisations.

The preliminary steps for developers of new bait vaccines for OVD include: selection of a candidate vaccine; determination of safety and efficacy in laboratory controlled trials; identification of a bait matrix well accepted by dogs; and evaluation of bait uptake in target populations. Responsible national authorities in countries contemplating OVD should choose a vaccine that has been thoroughly tested and through field studies select bait and delivery method best adapted to the local situation for successful access by dogs.

## Candidate vaccines for OVD

Several types of modified-live, attenuated or recombinant vaccines were evaluated for the oral rabies vaccination of dogs (OVD). SAD (Street-Alabama-Dufferin) Bern is a modified-live virus vaccine, cell-adapted derivative of the ERA (Evelyn Rokitnicki Abelseth) strain [19]. SAD B19 is a modified-live virus vaccine derived from the SAD Bern by selection [20, 21]. SAG2 (SAD Avirulent Gif) is a modified-live, attenuated rabies virus vaccine, selected from the SAD Bern strain in a two-step process of amino acid mutation using neutralizing monoclonal antibodies [22–24]. Vaccinia rabies glycoprotein (V-RG) is an attenuated (“modified-live”) recombinant vaccinia virus vector vaccine expressing the rabies virus glycoprotein gene [25–27]. AdRG1.3 is a human adenovirus-vectored recombinant vaccine containing a glycoprotein gene sequence from the ERA rabies virus strain [28]. CAV-2-E3Δ-RGP is a canine adenovirus-vectored recombinant vaccine with the rabies virus glycoprotein [29, 30]. rERAG333E is a recombinant ERA rabies virus strain containing a mutation from Arg to Glu at G333 position [31]. Other live-attenuated recombinant rabies virus vaccines (RV SN10-333; RV SPBN-Cyto c; RV SPBNGAS; RV SPBNGAS-GAS; VRC-RZ2) were also tested in dogs [26, 32, 33].

## Safety

The WHO recommends that the vaccines should be safe for humans, target species (including puppies) and for major endemic non-target species, likely to be attracted by the baits.

### In the target species

Safety requirements are more stringent for OVD than for oral vaccination of wildlife. In most situations, dogs are closely associated with human dwellings, activities and humans themselves. Particularly, since dogs under 3 months of age may form an important part of the local population in developing countries, and since there is a high probability of contact between young children and puppies, vaccines should not produce disease in such young dogs when administered *per os* or intramuscularly, at 10 times the dose used in the field [13]. The safety for the target species was evaluated in laboratory conditions and in indigenous dogs (Table 1) (to take into account the health status with parasitism, concurrent infections and/or nutritional deficiencies) orally and parenterally at doses up to 10^9.5^ tissue culture infective dose (TCID)_50_ for SAG2 [34], 10^9.6^ PFU (Plaque-Forming Unit) for V-RG [25] and 10^8.0^ PFU for SADB19 [35]. Both SAG2 and SAD B19 were also shown to be safe in dogs aged less than 10 weeks [35–37].

**Table 1:** **Safety**

Vaccine	Nb dogs	Age	Dog characteristics	Country	Administration	Dose per animal	Observation period (days)	Rabies virus positive at the end of the trial			Source
								Brains	Salivary glands	Salivary swabs	
SAG2	6		Laboratory dogs		Oral instillation	10^8.5^ TCID_50_	3–35	0		0	[13]
	6		Laboratory dogs		Brachial nerve plexus	10^8.5^ TCID_50_	3–35	0		0	
SAG2	21	< 3 months (N = 10 3–12 months (N = 3) > 1 year (N = 8)	Indigenous dogs	Tunisia	Oral instillation	10^9.5^ TCID_50_	90 or 180	0	0		[34]
SAG2	4	6–12 months	Laboratory dogs	India	DBL2 bait	10^8.5^ TCID_50_	219	0	0		[24]
	5 (immuno depressed)	6–12 months	Laboratory dogs		DBL2 bait	10^8.5^ TCID_50_	219	0	0		
	3	6–12 months	Laboratory dogs		Control	–	219	0	0		
SAG2	5	Adult	Laboratory beagles	CDC, US	Rabigen Oral	10^8.2^ TCID_50_	42–49	0			[23]
	3	Adult	Laboratory beagles		Rabigen Oral	10^7.5^ TCID_50_	42–49	0			
	5	Adult	Laboratory beagles		Rabigen Oral	10^7.4^ TCID_50_	42–49	0			
	5	Adult	Laboratory beagles		DBL2 bait	10^8.3^ TCID_50_	42–49	0			
	4	Adult	Laboratory beagles		DBL2 bait	10^7.2^ TCID_50_	42–49	0			
	4	Adult	Laboratory beagles		DBL2 bait	10^6.9^ TCID_50_	42–49	0			
SAG2	10	7–10 weeks	Indigenous dogs	South Africa	IM	10^9.0^ TCID_50_	120	0	0	8/10: positive 1 h post-admin, but not later	[36]
	10	7–10 weeks	Indigenous dogs	South Africa	Oral instillation	10^9.0^ TCID_50_	120	0	0	7/10:positive 1 h post-admin, but not later	
	1	7–10 weeks	Indigenous dogs	South Africa	Contact control	–	120	0	0		
SAD B19	8	< 10 weeks	Free-roaming indigenous dogs	Turkey	Oral instillation	2.4 × 10^7^ PFU	40–81	0			[35]
	2	< 10 weeks			Parenteral	2.4 × 10^7^ PFU	27–28	0			
	12	< 10 weeks			Oral instillation	4.2 × 10^8^ PFU	24–97	0			
	6	< 10 weeks			Parenteral	10^8^ PFU	30–69	0			
SAD B19	4	6 months	Free-roaming indigenous dogs	Turkey	IC	2.5 × 10^7^ PFU (4th passage IC in foxes)	40				[35]
V-RG	6		Indigenous dogs	Tunisia	Oral instillation	10^8.5^ TCID_50_	123				[78]
	6				Oral instillation	10^9.5^ TCID_50_	123				
VRC-RZ2	6	3–12 months	Indigenous dogs	Kazakhstan	Bait	10^7.7^ TCID_50_	20			0/6 tested 5 and 10 days post-adm	[33]

DBL2: dog bait lyophilized (freeze-dried SAG2 bait), IC: intracerebrally, IM: intramuscularly, PFU: plaque forming units, TCID_50_: median tissue culture infectious doses.

During the oral rabies vaccination campaigns in Finland from 2011 to 2014, 160 000 to 360 000 SAG2 vaccine baits were distributed annually. There were nine reports from dog owners or veterinarians about dogs experiencing signs associated with the consumption of SAG2 baits during hunting [38]. Reported gastrointestinal signs (e.g., vomiting, inappetence, constipation or diarrhea) were probably related to the ingestion of the aluminium/polyvinyl chloride sachet and behavioral signs (e.g., restlessness, listlessness and unwillingness to continue hunting) were probably related to the discomfort caused by the ingestion of multiple baits [38].

### In non-target species

No harmful effects should occur when local wild and domestic animal species that may consume baits are given orally a dose of vaccine equivalent to 10 times the field concentration. In addition, vaccine safety should be demonstrated in common local rodents when they are given the field dose of vaccine orally and intramuscularly [13]. The candidate live vaccine should also be tested for safety in non-human primates, such as chimpanzees, baboons, rhesus monkeys, etc. [6].

The safety for non-target species, including non-human primates, was extensively demonstrated for SAG2 [4], V-RG [5] and SAD B19 [39]. After oral administration to immunocompromised animals, V-RG (tested on mice and cats) did not induce adverse events [5]. In contrast, SAD B19 induced rabies in nude mice (2 out of 6 mice) [39]. Residual pathogenicity of SAD B19 was also reported in wild rodents (5.7% of wild rodents receiving orally a field concentration died of rabies), as described [39]. In addition, adverse events were observed after administration of SAD Bern to wild rodents [40], wild and domestic carnivores [41] and baboons [42] but not to rhesus macaques [43].

### Virus excretion

Humans may be exposed to the vaccine through contact with a freshly vaccinated dog (e.g. by licking or biting). The possibility of excretion of vaccine virus in the saliva of vaccinated animals should be examined, since excretion may be indicative of local replication and consequently an increased risk of mutation, reversion to pathogenicity and transmission. The virus replication should be limited temporally and quantitatively and any virus recovered characterized [6]. SAG2 virus is cleared rapidly after ingestion. Following oral administration of concentrated vaccine suspension ranging from 10^8.3^ to 10^9.0^ TCID_50_, SAG2 virus was detected occasionally in saliva for about 1 h after ingestion in indigenous and laboratory dogs, but not later up to 5 days [22, 24, 36, 37, 44]. SAG2 virus was detected in tonsils and buccal mucosa from dogs 24 to 96 h after oral administration, indicating a local replication in the tonsils [44]. No SAD B19 virus was detected in saliva from puppies collected from 2 to 72 h after oral administration of a dose of 4.2 × 10^8^ PFU [35]. Residual V-RG was found in oral swabs at 1 h after oral instillation of a dose of 10^8^ TCID_50_ or 10^9^ TCID_50_ of the vaccine in approximately half of the dogs [13]. The SAD Bern virus was detected in salivary samples on day 3 in a dog instilled with a dose of 10^7.5^ TCID_50_ [19]. The SPBNGAS-GAS virus was detected in saliva swabs from 3 out of 12 tested dogs taken 4 h after oral administration of a dose of 10^9.1^ focus forming unit (FFU)/mL [45]. Future research should be targeted to determining dynamics of oral vaccination, i.e. the primary sites of viral replication and the rapidity of clearance of candidate vaccines by using standardised procedures.

### Reversion to virulence studies

The absence of reversion to virulence was demonstrated after different passages in mice and/or target species (fox) for SAG2, V-RG and SAD B19 [4, 5, 39].

### Human exposure

An effective pharmacovigilance system should be established to detect any possible human exposure to vaccine [13]. Humans accidentally in contact with the vaccine (by mouth, nose, eye or wound) should receive rabies post-exposure prophylaxis, except for V-RG for which pre-exposure or post-exposure rabies vaccination is not recommended, since this vaccine does not include infectious rabies virus [6].

During small scale studies in Tunisia, human exposure risks differed according to the mode of distribution. A total of 25 human unprotected contacts with baits were observed for 314 baits distributed according to a dog owner participation-based delivery system, or ~1 contact per 12.5 baits distributed [46], compared to 32 contacts for 1168 baits placed along transect-lines according to the wildlife immunisation model (WIM), at ~1 contact per 36.5 baits distributed [47]. These bait matrix contact cases did not result in a post-exposure prophylaxis. Approximately 250 million V-RG doses have been distributed globally since 1987 without any reports of adverse reactions in wildlife or domestic animals [5]. Only two human exposures, from contact with vaccinated dogs, resulted in vaccinia virus infections in a pregnant woman with epidermolytic hyperkeratosis and in a woman receiving immunosuppressive medications for inflammatory bowel disease [5].

World Health Organisation recommended only vaccines with the lowest known residual pathogenicity in experimental conditions and in the field, such as SAG2 and V-RG, for OVD [48]. SAD Bern elicited a strong antibody response in vaccinated dogs and complete protection against rabies after direct instillation into the mouth, but safety concerns in wild rodents [40] and non-human primates [42] and the detection of virus in the saliva of dogs 3 days after vaccination prevented its use for OVD [19].

## Efficacy

Efficacy is defined as protection of a vaccinated target animal after live virus challenge. The challenge virus should preferably be a well-characterized street virus of dog origin (ideally of salivary gland material). A concentration sufficient to cause rabies in at least 80% of controls should be administered. Immunogenicity measured by antibody response is considered as only a part of the measurement of efficacy [13]. The efficacy of an oral vaccine candidate should be assessed when administered by oral instillation, and when given in a bait to caged dogs (vaccine-in-bait efficacy). Moreover, efficacy of vaccine baits should be studied in the field [6].

Different rabies vaccine candidates were shown to induce rabies virus neutralising antibodies (VNA) when administered orally to dogs: SAG2 [22–24], V-RG [25–27, 49], SAD Bern [19], SAD B19 [20, 21], AdRG1.3 [28], CAV-2-E3Δ-RGP [30], rERAG333E [31], and different live-attenuated recombinant rabies virus vaccines (RV SN10-333; RV SPBN-Cyto c; RV SPBNGAS; RV SPBNGAS-GAS) [26, 32].

Efficacy, as defined above, was demonstrated in dogs after direct oral instillation of a vaccine suspension for SAG2 [22, 23], V-RG [25], SAD Bern [19], SAD B19 [20], and different live-attenuated recombinant rabies virus vaccines (RV SN10-333; RV SPBN-Cyto c; RV SPBNGAS; RV SPBNGAS-GAS) [26].

Some vaccine strains induced protection against rabies virus challenge after administration into a bait (Additional file 1). Most studies have been conducted using SAG2 freeze-dried baits distributed to either laboratory dogs or indigenous Tunisian or Indian dogs which were challenged up to 180 days post-baiting [22–24, 34, 44, 50]. This vaccine has been registered for the control of canine rabies in India in 2006 [24] and is, to our knowledge, the sole oral rabies vaccine registered for dogs and wildlife. When administered into baits used for oral immunisation of wildlife, V-RG induced protection against rabies in laboratory dogs [27]. SAD B19 distributed with Köfte baits or boiled intestine baits to free-roaming indigenous Turkish dogs induced protection [20, 21]. CAV-2-E3Δ-RGP induced a complete protection against rabies 15 weeks after administration in a bait to indigenous Chinese dogs [30].

Lastly, WHO recommends evaluation of the vaccine-bait efficacy in terms of duration of immunity under laboratory and field conditions. For wildlife, regulatory authorities require that the assessment of the efficacy of oral vaccines should be demonstrated in the target species at least 6 months after administration of the vaccine bait using 25 vaccinated and 10 control animals [51]. According to European Pharmacopoeia and the US Code of Federal Regulations, efficacy of inactivated vaccines for dogs must be demonstrated by challenge at the end of the immunity period claimed by the manufacturer (generally 1 year using 25 vaccinates and 10 controls). Three oral vaccine candidates have been evaluated in dogs with a challenge performed 6 months (SAG2 [22] and VRC-RZ2 [33]) and 2 years (CAV-2-E3Δ-RGP [30]) after vaccine bait administration.

Efficacy studies have confirmed that rabies VNA are a critical immune effector and generally correlated with protection. However, resistance to rabies virus challenge was reported in some dogs despite any detectable VNA after vaccination with SAG2 [23, 24, 44], V-RG [26, 27] and different recombinant rabies virus vaccines [26]. An anamnestic response was evident after rabies virus challenge in most dogs vaccinated with SAG2, V-RG and SAD B19, including dogs which did not develop VNA after primary vaccination [21, 23, 26]. The lack of detectable antibodies in a fraction of dogs after oral rabies vaccination may cause recurring problems in the field, because there is still no reliable predictive marker of vaccine protection [27].

The CAV-2-E3Δ-RGP candidate showed promising results after administration in a bait. A large proportion (88%) of dogs immunised orally developed VNA, which persisted for 2 years in 80% of dogs. All 10 of 10 dogs survived a rabies virus challenge performed 2 years after their vaccination [30]. However, Wright et al. showed that pre-existing antibodies against CAV, naturally occurring in South Africa, inhibited the development of VNA against rabies in dogs immunized with CAV-2-E3Δ-RGP. All dogs, except one which received prior vaccination against CAV and were then immunized with CAV-2-E3Δ-RGP orally, developed rabies after challenge [52].

## Bait development and evaluation of preferences

Bait candidates should be tested both in owned dogs living in the households within the area (or country) where oral vaccination is to be applied, and in ownerless and free-roaming owned dogs [6]. Ideally, baits should be produced locally in large quantities and as inexpensively as possible. Field trials should be conducted to compare machine-manufactured versus hand-crafted baits [6]. Standardized protocols for testing bait preferences have been described by Linhart [53, 54] and the WHO [6]. Several parameters should be tested and optimized, such as bait palatability, shape, size, and texture of the bait matrix, as well as the blister, to enable an efficient release of the vaccine in the oral cavity [55].

Many studies have been conducted in different countries to evaluate bait preferences in dogs (Additional file 2). Bait uptake was evaluated by the use of topical (e.g., methylene blue, rhodamine B) [56, 57] or systemic markers, such as sulfadimethoxine [46], by means of a tracking-station or by direct observation. Data collected included overall bait acceptance, proportion of baits consumed, speed of bait consumption, proportion of baits swallowed, and proportion of blisters (containing vaccine or placebo) punctured and/or discarded. Baits tested were either made locally or manufactured using different flavours. These studies documented clear regional differences which could be related to different food preferences, lack of familiarity with the material proposed and different experiences of the dog population. Local-made baits with the highest acceptance rates were chicken-heads in Tunisia [56, 58] and Guatemala [59], the Köfte-bait (minced meat mixed with bread crumbs) in Turkey [60], boiled pig intestines in the Philippines [61], or boiled bovine intestine at the US Indian Navaro Nation reservation [55]. In Turkey, chicken-head baits were less accepted by dogs in urban areas of Istanbul mainly fed with household leftovers and offal [62]. Dog biscuits were preferred in Mexico [63]. Manufactured baits, such as fish-meal polymers baits or coated sachets used for vaccination of wildlife, were well accepted in Sri Lanka [64], in Tunisia [56], on US Indian reservations, such as the Navajo and the Hopi Nation lands [65], in Indonesia [14] and in Thailand [14], but less preferred by livestock guardian dogs in Israel [66], in Egypt [54] or Turkey [62]. Poultry-flavoured baits were preferred in Guatemala [59], bacon-flavoured baits on the Navaro Nation [28, 67], and liver-flavoured baits in Thailand [14]. Human preferences should be taken into account during such trials. For instance, in the Philippines, dog owners were reluctant to distribute chicken-head baits to their dogs, due to a concern they could develop a preference for free-roaming poultry [21].

Results of bait acceptance cannot be compared through the various studies due to different test methodologies, bait types and cultural environments. Interestingly, high bait acceptance rates do not necessarily mean that vaccination is successful, as illustrated by the Köfte bait, which was often completely swallowed without being chewed on, including the vaccine container used [60].

Overall, these studies show that there is no universal bait for dogs. The most inexpensive approach for developing countries with limited financial resources would be to incorporate imported vaccine-loaded blisters in locally-produced baits.

## Thermostability

Vaccine thermostability in the bait under field conditions needs to be assessed. The stability of V-RG and SAG2 vaccines in baits used in wildlife was extensively demonstrated in various environmental conditions, including tropical climates [4, 5]. Provided that the cold chain is maintained during transportation and storage, thermostability is less crucial for OVD than for wildlife, as baits are distributed to dogs either directly by hand or placed in selected sites, with unconsumed baits being recovered within 24 h.

## Bait delivery

Different delivery systems were evaluated in the field: (i) distribution of baits to owned dogs via their owner collecting the bait from a central location; (ii) door to door baiting; (iii) placement of baits at selected sites where they are accessible to free-roaming dogs (the so-called wildlife-immunisation model (WIM); and (iv) distribution of baits to dogs encountered in the street (the so-called “hand-out model”). The first system of distribution reaches primarily owned dogs accessible to parenteral vaccination, and to a lesser extent, owned dogs inaccessible to parenteral vaccination. This method was successfully used in Tunisia [46]. Such a method involving dog owners would however necessitate modifications of regulation on the delivery and application of veterinary rabies vaccines currently enforced in many countries. Door to door baiting enables safe administration of vaccine baits to owned dogs, but is time consuming [47] and the behaviour of the vaccination team may influence bait acceptance. For example, some animal health technicians consistently achieve better acceptance rates than others [68]. The WIM model enables vaccination of free-roaming and feral dogs which represent a potentially higher risk group in terms of rabies virus transmission [6, 69]. In WIM model, baits should be preferably distributed in late afternoon/early evening and baits not consumed should be collected within 18–24 h. The WIM model gave encouraging results when baits were distributed at selected sites, where large numbers of community dogs congregate, such as slaughterhouses, garbage dumps or public markets and along paths frequently used by dogs, as reported in Morocco, with up to 73% baits disappeared overnight [70], Tunisia, where 40% baits disappeared within 24 h [47] and Turkey, where on average, ~50% of Köfte-baits disappeared within 270 min [71]. However, the risks of human contact with the bait delivered using the WIM model, especially of children, are to be evaluated before large application, as well as the probability of unintentional contacts with non-target species (bait competitors). For instance, when placebo Köfte-baits were placed at selected sites in urban areas from Turkey, crows located 30% of the baits during the day, while at night cats took up to 27% of the baits. In addition, free-roaming owned dogs already vaccinated against rabies by the parenteral route can compete for distributed baits [71]. In contrast, the risk of unintentional exposure to non-target species is limited with the three other distribution models. In the “hand-out model”, all owned restricted dogs that cannot be handled by their owners and all free-roaming dogs encountered in the streets are offered a bait. This model was used successfully in Morocco [70], in Turkey [60], in the US on the Navajo Nation reservation [55, 65], the Philippines [72] and Guatemala [59].

Importantly, the choice of the methods of distribution requires adaptation to the local situation and should be incorporated in the rabies control programme as a complement to parenteral vaccination. Factors that help determine the most appropriate bait and delivery systems in a given area include acceptance rates by target and non-target species, socio-cultural acceptance and efficacy of the delivery system, human density, dog population structure and dynamics, feeding patterns and the economics associated with programme implementation [6]. Information on dog ecology and dynamics is important, such as the proportion of owned (with their degree of confinement) and ownerless dogs, the annual dog population’s turn-over, the proportion of dog populations accessible for parenteral versus oral vaccination, and the size of the dog population [6]. For instance, the “hand-out model” is better adapted in Asia, since free-roaming dogs are usually people friendly and more easily approached, whereas in Maghreb, dogs are often wary and easily scared as many people, afraid of dog bites and diseases, avoid contacts by throwing stones at them [70]. Bishop reported that in South Africa, approximately 86% of the dog owners preferred oral vaccination to the parenteral route [68]. In particular, there is a general reluctance in Kwazulu-Natal (South Africa) communities to bring hunting-type dogs (greyhounds and whippets) for parenteral vaccination [68]. In certain islands of the Indonesian archipelago, such as West Sumatra and Kalimantan, accessibility of dogs to parenteral vaccination is low, due to cultural and religious beliefs and misconceptions about side effects of parenteral vaccination [14].

The vaccination coverage should reach at least 70% of the total dog population to successfully eliminate rabies [73, 74]. Different studies showed that overall vaccination coverage was increased to levels assumed to stop the spread of rabies through the use of OVD. In Sri Lanka, the vaccination coverage obtained after a parenteral vaccination campaign of dogs in households was increased from 63 to 78% by use of a supplementary oral vaccination campaign [64]. During house-to-house campaigns conducted in Istanbul Turkey, vaccination coverage was increased by 18 to 21% after distribution of baits to owned dogs that were inaccessible to parenteral vaccination, achieving an overall vaccination coverage of 74 to 84% [75]. In another study in the Philippines, none of the owned dogs had been vaccinated against rabies previously, and all, except one dog, were free-roaming. During the vaccination campaign, only 8% of the dogs could be restrained and vaccinated by oral instillation. Other dogs encountered were offered boiled intestine vaccine baits. The vaccination coverage of the dog population (> 2 months old), including dogs vaccinated by instillation or with a bait, reached 76% [72].

## Cost/effectiveness

Experiments were carried out in Tunisia to estimate and compare costs of the different bait distribution methods. Compared to door to door distribution (34.3 person min per bait, US$ 3.9/dog accepting a bait) and transect line baiting (47.9 person min per bait, US$ 18.0/dog accepting a bait), distribution to dog owners at a central location was the most cost-effective, at 7.6 person min per bait, US$ 1.6–1.8/dog accepting a bait [46, 47]. However, the dog populations targeted by each of these methods of distribution are different. Even if the WIM model is more expensive and reaches a lower proportion of the population, it could be the only way to vaccinate truly feral and many unsupervised owned and ownerless community dogs.

## Limitations

Although the application of OVD may provide significant overall benefits to the mass vaccination used routinely towards the elimination of canine rabies on a global basis, there are a number of potential issues that are quite distinct between the two strategies. Although it may become technically more feasible in the future to provide purified, highly immunogenic antigens for oral rabies vaccination, currently all biologics involved in the OVD are self-replicating agents. As such, the relative safety and potential severity of adverse events is different than with inactivated vaccines that are administered by injection. When vaccines are administered by injection, greater control is involved with delivery to a single animal as contrasted to deliberate environmental distribution in a bait over time. Moreover, many of the modern recommended biologics for OVD are recombinant products and as such may involve different regulatory standards applicable to genetically-modified organisms intended for environmental release. In addition, due in part to the method of production, the costs of inactivated parenteral vaccines are much less expensive than those of biologics used thus far for OVD. To date, all countries that have eliminated the transmission of canine rabies virus have used mass parenteral vaccination only [76]. The more routine use of OVD on large scales as an adjunct to traditional canine vaccination will allow a proper assessment of this method particularly in regards to its efficacy and cost–benefit advantages.

## Conclusions

Oral vaccination has the potential to become an important adjunct to traditional rabies prevention and control measures, such as parenteral vaccination of dogs, control of stray dog populations, public education and human rabies prophylaxis [23]. Many vaccine strains have been thoroughly evaluated for OVD, and at least two (SAG2 and V-RG vaccines) have currently been shown to be both safe and efficacious. Different types of baits and baiting strategies have been developed and can be used to target certain categories of dogs.

Field trials have shown the potential benefit of OVD in different countries and socio-economical settings, such as Tunisia [46, 47], Turkey [75], Sri Lanka [64], the Philippines [72] the Republic of South Africa [68], and Morocco [70]. The use of OVD increases the overall number of vaccinated dogs (quantitative effect), including a significant proportion of free-roaming owned and ownerless dogs playing an important role in the disease maintenance and spread in rabies infected areas (qualitative effect) [75]. Safety for target and non-target species, including humans, of recommended vaccines and bait distribution strategies has been studied extensively [6–14].

Since dogs are very closely associated with humans, especially with children, in a majority of cultures, the likelihood of direct exposure and of passive vaccine virus transfer to humans is considerably higher for OVD than for wildlife immunization programmes [13]. Different situations may lead to human exposure. Some have been shown to occur at a low rate, e.g., exposure to the vaccine contained in a bait by direct contact. Other scenarios, such as exposure through licking of mucosa or biting by a freshly vaccinated dog are possible but must be rare. Finally, the hypothetical exposure to a vaccine virus reverting to increased virulence after passaging into immunosuppressed target or non-target species is highly unlikely, considering what we know about recommended vaccines. Consequently, national regulatory authorities should assess these results and balance risks of vaccine-induced untoward events associated with improbable scenarios and the real risk of contracting the disease following contact with a naturally infected dog. The increase in the immunisation coverage resulting from the wise application of OVD may be crucial to achieve rabies elimination.

Cost-wise, countries should realize that when targeting certain high risk components of the dog population, such as feral and free-roaming dogs, the cost per dog vaccinated by the oral route will be higher than that established for a parenteral vaccination [6, 46, 47].

Countries where OVD will be used in combination with parenteral vaccination should follow and adapt, whenever necessary, to local conditions established and standardized methodologies to evaluate these vaccines and their applications. Table 2 proposes a list of the main criteria that should be considered for assessing oral rabies vaccines for use in dogs.

**Table 2:** **Prerequisites and criteria for oral vaccines in dogs**

Efficacy of the vaccine candidate	Direct oral instillation of the vaccine suspension to laboratory dogs Administration of the vaccine bait to confined dogs Distribution of the vaccine bait in the field
Safety of the vaccine candidate	In the target species (10 times the field dose) In non-target species (local wild and domestic animals) (10 times the field dose) In wild rodents (field dose) In non-human primates (10 times the field dose) In immunodeficient laboratory animal model (oral, intracerebral, intramuscular routes) Excretion in the saliva of puppies Reversion to virulence studies
Bait candidate	Preference in owned dogs living in the households within the area (or country) where oral vaccination is to be applied Preference in ownerless and free-roaming owned dogs Thermostability

Several countries, especially those which have been fighting canine rabies for decades and are today close to achieving the goal of elimination (such as Thailand, Sri Lanka, Mexico, Brazil, etc.) [76], should use modern tools to reach those dog subpopulations which have been escaping parenteral vaccination for years. These countries should seriously consider OVD as a complementary measure to mass parenteral vaccination campaigns to increase vaccination coverage, by using manufactured baits or by producing vaccine locally and adopt a specially flavoured existing bait or use locally-produced baits most adapted to local dog preferences. The time is now, for much wider application of OVD in developing countries to help achieve the Global initiative Zero human case of rabies by 2030 [77].

## Additional files

**Additional file 1.** **Efficacy studies.** This table contains compiled information summarizing efficacy data in oral rabies vaccines for dogs studies.

**Additional file 2.** **Attractiveness studies.** This table contains compiled information summarizing attractiveness data on oral rabies vaccines for dogs studies.

## Abbreviations

CAV: canine adenovirus
DBL2: freeze-dried SAG2 bait
DM: sulfadimethoxine
ERA: Evelyn Rokitnicki Abelseth
FAVNT: fluorescent antibody virus neutralisation test
FFU: focus forming unit
IC: intracerebrally
IM: intramuscularly
MNT: mouse neutralisation test
OIE: World Organisation for Animal Health
OVD: oral vaccination of dogs
PFU: plaque-forming unit
PVC: polyvinyl chloride
RFFIT: rapid fluorescent foci inhibition test
SAD: Street-Alabama-Dufferin
SAG: Street Alabama Gif
SPBN: second generation rabies virus based vaccine
TCID: tissue culture infective dose
VNA: virus neutralising antibody
V-RG: vaccinia rabies glycoprotein
WHO: World Health Organisation
WIM: wildlife immunisation model
