# Correction to: Oral vaccination of dogs: a well-studied and undervalued tool for achieving human and dog rabies elimination

**DOI:** 10.1186/s13567-018-0579-x

**Published:** 2018-08-30

**Authors:** Florence Cliquet, Anne-Laure Guiot, Michel Aubert, Emmanuelle Robardet, Charles E. Rupprecht, François-Xavier Meslin

**Affiliations:** 1 French Agency for Food, Environmental and Occupational Health & Safety (ANSES), Nancy Laboratory for Rabies and Wildlife, European Union Reference Laboratory for Rabies, European Union Reference Laboratory for Rabies Serology, OIE Reference Laboratory for Rabies, WHO Collaborating Centre for Research and Management in Zoonoses Control, Technopôle agricole et vétérinaire de Pixérécourt, CS 40009, 54220 Malzéville, France; 2 Conseils en Pharmacie et Biologie, 2 place des Quatre Vierges, 69110 Sainte Foy les Lyon, France; 3 1088 Chemin des Maures, 83440 Callian, France; 4 LYSSA LLC, Lawrenceville, GA USA; 5 Le Grand-Saconnex, Switzerland

## Correction to: Vet Res (2018) 49:61 http://doi.org/10.1186/s13567-018-0554-6

The original article [1] contained an error in the Author details paragraph. “^5^Neglected Zoonotic Diseases, World Health Organization, Geneva, Switzerland” should be replaced by “^5^Le Grand-Saconnex, Switzerland”.
