# Supplementary material for: Oral vaccination of dogs: a well-studied and undervalued tool for achieving human and dog rabies elimination
Source: Vet Res. 2018 Jul 13;49:61. doi: 10.1186/s13567-018-0554-6 (PMC6045873; doi:10.1186/s13567-018-0554-6)
Supplement: Supplementary file 1 — Additional file 1. Efficacy studies. This table contains compiled information summarizing efficacy data in oral rabies vaccines for dogs studies. [file 13567_2018_554_MOESM1_ESM.docx]

Additional file 1: Efficacy DBL2: freeze-dried SAG2 bait , FAVNT: Fluorescent Antibody Virus Neutralisation Test, MNT: Mouse Neutralisation Test, ND: Not Documented, PFU: Plaque Forming Units, RFFIT: Rapid Fluorescent Foci Inhibition Test , TCID50: median Tissue Culture Infectious doses

| **Vaccine** | **Nb dogs** | **Age and dog characteristics** | **Rabies status on D0** | **Country** | **Route of administration** | **Dose per animal** | **Ab response throughout the pre-challenge period** | | | | **Challenge** | **Survival** | **Duration follow-up** | **Rabies virus** | **Source** |
| --- | --- | --- | --- | --- | --- | --- | --- | --- | --- | --- | --- | --- | --- | --- | --- |
|  |  |  |  |  |  |  | **Test** | **Threshold positivity** | **Seropositivity** | **≥ 0.5 IU/mL** | **after vaccination** | **after vaccination** | **after challenge** | **positive brains** |  |
| SAG2 | 7 | < 3 months (N=1) 3-12 months (N=1)  > 1 year (N=5) mongrel indigenous dogs | Never vaccinated against rabies | Tunisia | DBL2 bait | 10^8.3^ TCID_50_ | FAVNT | 0.24 IU/mL | 2/7 | 2/7 | 33 days | 5/7 | 160 days | 2/7 | [34] |
|  | 6 | < 3 months (N=4)  3-12 months (N=2) mongrel indigenous dogs | Never vaccinated against rabies |  | Control | - |  |  | 0/6 | 0/6 | 33 days | 1/6 | 160 days | 5/6 |  |
| SAG2 | 9 | 6-12 months indigenous stray dogs | Seronegative on D0 | India | DBL2 bait | 10^8.5^ TCID_50_ | FAVNT | 0.24 IU/mL | 6/9 | 5/9 | 109 days | 9/9 | 90 days | 0/9 | [24] |
|  | 5 | 6-12 months indigenous stray dogs | Seronegative on D0 |  | Control | - |  |  |  |  | 109 days | 0/5 | 90 days | 5/5 |  |
| SAG2 | 4 | 5-6 months old laboratory beagles | Seronegative on D0 | France | DBL2 bait | 10^8.0^ TCID_50_ | FAVNT |  | 0/4 |  | 29 days | 3/4 |  |  | [50] |
|  | 4 | 5-6 months | Seronegative on D0 |  | DBL2 bait | 10^9.0^ TCID_50_ |  |  | 0/4 |  | 29 days | 3/4 |  |  |  |
|  | 2 | 5-6 months | Seronegative on D0 |  | Control | - |  |  | 0/2 |  | 29 days | 0/2 |  |  |  |
| SAG2 | 5 | Laboratory adult beagles | Seronegative on D0 | CDC, US | Rabigen Oral (liquid) | 10^8.2^ TCID_50_ | RFFIT | Complete neutralization at 1:5 | 4/5 | 2/5 | 6-7 weeks | 4/5 | ≥ 90 days | 1/5 | [23] |
|  | 3 | Laboratory adult beagles | Seronegative on D0 |  | Rabigen Oral | 10^7.5^ TCID_50_ |  |  | 1/3 | 1/3 | 6-7 weeks | 2/3 | ≥ 90 days | 1/3 |  |
|  | 5 | Laboratory adult beagles | Seronegative on D0 |  | Rabigen Oral | 10^7.4^ TCID_50_ |  |  | 3/5 | 0/5 | 6-7 weeks | 3/5 | ≥ 90 days | 2/5 |  |
|  | 5 | Laboratory adult beagles | Seronegative on D0 |  | DBL2 bait | 10^8.3^ TCID_50_ |  |  | 4/5 | 3/5 | 6-7 weeks | 5/5 | ≥ 90 days | 0/5 |  |
|  | 4 | Laboratory adult beagles | Seronegative on D0 |  | DBL2 bait | 10^7.2^ TCID_50_ |  |  | 3/4 | 0/4 | 6-7 weeks | 4/4 | ≥ 90 days | 0/4 |  |
|  | 4 | Laboratory adult beagles | Seronegative on D0 |  | DBL2 bait | 10^6.9^ TCID_50_ |  |  | 1/4 | 0/4 | 6-7 weeks | 1/4 | ≥ 90 days | 3/4 |  |
|  | 5 | Laboratory adult beagles | Seronegative on D0 |  | Control | - |  |  |  |  | 6-7 weeks | 0/5 | ≥ 90 days | 5/5 |  |
| SAG2 | 10 | 1.5-2.5 years old laboratory beagles | Never vaccinated against rabies | CDC, US | Bait | 10^8.0^ TCID_50_ | RFFIT | Complete neutralization at 1:5 | 8/10 |  | 180 days | 10/10 | ≥ 90 days | 0/10 | [22] |
|  | 10 | 1.5-2.5 years old laboratory beagles | Never vaccinated against rabies |  | Bait | 10^9.0^ TCID_50_ |  |  | 10/10 |  | 180 days | 10/10 | ≥ 90 days | 0/10 |  |
|  | 10 | 1.5-2.5 years old laboratory beagles | Never vaccinated against rabies |  | Oral instillation | 10^8.0^ TCID_50_ |  |  | 9/10 |  | 180 days | 10/10 | ≥ 90 days | 0/10 |  |
|  | 10 | 1.5-2.5 years old laboratory beagles | Never vaccinated against rabies |  | Oral instillation | 10^9.0^ TCID_50_ |  |  | 10/10 |  | 180 days | 10/10 | ≥ 90 days | 0/10 |  |
|  | 10 | 1.5-2.5 years old laboratory beagles | Never vaccinated against rabies |  | Control | - |  |  | 0/10 |  | 180 days | 2/10 | ≥ 90 days |  |  |
| SAG2 | 12 | Adult laboratory beagles | Never vaccinated against rabies | CDC, US | DBL2 bait | 10^8.3^ TCID_50_ | RFFIT | Complete neutralization at 1:5 | 4/12 |  | 4 weeks | 10/12 | ≥ 90 days | 2/12 | [44] |
|  | 6 | Adult laboratory beagles | Never vaccinated against rabies |  |  |  |  |  | 0/6 |  |  | 0/6 | ≥ 90 days | 6/6 |  |
| V-RG | 6 | Beagles > 6 months | Never vaccinated against rabies | CDC, US | Oral instillation | 10^8.9^ TCID_50_ | RFFIT | Complete neutralization at 1:5 | 4/6 |  | 35 days | 5/6 | ≥ 90 days | 1/6 | [26] |
| SN10-333 | 6 | Beagles > 6 months | Never vaccinated against rabies |  | Oral instillation | 10^8.6^ TCID_50_ |  |  | 6/6 |  | 35 days | 6/6 | ≥ 90 days | 0/6 |  |
| SPBN-Cyto c | 6 | Beagles > 6 months | Never vaccinated against rabies |  | Oral instillation | 10^8.4^ TCID_50_ |  |  | 5/6 |  | 35 days | 6/6 | ≥ 90 days | 0/6 |  |
| SPBNGA | 6 | Beagles > 6 months | Never vaccinated against rabies |  | Oral instillation | 10^8.2^ TCID_50_ |  |  | 4/6 |  | 35 days | 6/6 | ≥ 90 days | 0/6 |  |
| SPBNGA-GA | 6 | Beagles > 6 months | Never vaccinated against rabies |  | Oral instillation | 10^8.6^ TCID_50_ |  |  | 5/6 |  | 35 days | 6/6 | ≥ 90 days | 0/6 |  |
|  | 12 | Beagles > 6 months | Never vaccinated against rabies |  | Control | - |  |  | 0/12 |  | 35 days | 0/12 | ≥ 90 days | 12/12 |  |
| V-RG | 4 | Beagles > 6 months | Never vaccinated against rabies | France | Oral instillation | 10^8.6^ PFU | RFFIT |  | 1/4 | 1/4 | 69 days | 0/4 |  |  | [25] |
|  | 4 | Beagles > 6 months | Never vaccinated against rabies |  | Oral instillation | 10^9.6^ PFU |  |  | 4/4 | 4/4 | 69 days | 4/4 |  |  |  |
|  | 5 | Beagles > 6 months | Never vaccinated against rabies |  | Control |  |  |  |  |  | 69 days | 0/5 |  |  |  |
| V-RG | 9 | Beagles: 9-11 months | Seronegative on D0 | France | Rectangular bait | 10^8.4^ TCID_50_ | FAVNT |  |  | 3/7 | 30 days | 5/7 | 90 days | 2/7 | [27] |
|  | 9 | Beagles: 9-11 months | Seronegative on D0 |  | Square bait | 10^8.4^ TCID_50_ |  |  |  | 1/9 | 30 days | 8/9 | 90 days | 1/9 |  |
|  | 3 | Beagles: 9-11 months | Seronegative on D0 |  | Rectangular placebo bait | - |  |  |  | 1/3 | 30 days | 0/3 | 90 days | 3/3 |  |
|  | 3 | Beagles: 9-11 months | Seronegative on D0 |  | Square placebo bait | - |  |  |  | 0/3 | 30 days | 0/3 | 90 days | 3/3 |  |
| V-RG | 6 | Indigenous dogs | ND | Tunisia | Oral instillation | 10^8.5^ TCID_50_ | RFFIT | 0,5 IU/mL | ND ; Mean of 0.15 IU/mL at D21 |  | 123 days | 5/6 |  |  | [78] |
|  | 6 | Indigenous dogs | ND |  | Oral instillation | 10^9.5^ TCID_50_ |  |  | ND ; Mean of 1.1 IU/mL at D21 |  | 123 days | 6/6 |  |  |  |
|  | 5 | Indigenous dogs | ND |  | Control | - |  |  | ND ; Mean of 0 IU/mL at D21 |  | 123 days | 0/5 |  |  |  |
| V-RG | 400 | Indigenous dogs | Seronegative on D0 | Thailand | Bait | ND | ND | > 0.1 IU/mL | 50% on D120, 71% on D180, 58% on D360 |  |  |  |  |  | [49] |
|  |  | Indigenous dogs | Seropositive on D0 (85%) |  |  |  |  |  | 92% on D30, 86% on D120, 98% on D180, 88% on D360 |  |  |  |  |  |  |
| SAD B19 | 8 | Free-roaming indigenous dogs | Seronegative on D0 | Turkey | Oral instillation | 3.0 x 10^7^ PFU | RFFIT |  |  | 3/8 |  |  |  |  | [20] |
|  | 7 | Free-roaming indigenous dogs | Seronegative on D0 |  | Oral instillation | 1.5 x 10^8.5^ PFU |  |  |  | 6/7 |  |  |  |  |  |
| SAD B19 | 23 | Owned dogs | ND | Turkey | Köfte-bait | 3.0 x 10^7^ PFU | RFFIT |  |  | 6/23 |  |  |  |  | [20] |
| SAD B19 | 16 | Free-roaming indigenous dogs | Seronegative on D0 | Turkey | Köfte-bait | 2.1 x 10^7^ PFU | RFFIT |  |  | 3/16 | 74 days | 9/16 |  |  | [20] |
| SAD B19 | 6 | 3-10 months free-roaming indigenous dogs | Seronegative on D0 | Turkey | Köfte-bait | 10^8.2^ PFU | RFFIT |  | 5/6 | 5/6 | 57 days | 5/6 | 72 days | 1/6 | [21] |
|  | 6 | 3-10 months free-roaming indigenous dogs | Seronegative on D0 |  | Intestine bait | 10^8.2^ PFU |  |  | 5/6 | 4/6 | 57 days | 5/5 | 72 days | 0/5 |  |
|  | 6 | 3-10 months free-roaming indigenous dogs | Seronegative on D0 |  | Control | - |  |  |  |  | 57 days | 0/6 | 72 days | 6/6 |  |
| SAD B19 | 14 | > 2 months owned dogs | ND | The Philippines | Boiled intestine bait | 10^8.4^ PFU | RFFIT |  |  | 10/14 |  |  |  |  | [72] |
| AdRG1.3 | 26 | Feral dogs | Various status | Navaro nation facility, U.S. | Bacon flavored Ultralite bait | > 10^9.75^ TCID_50_ | RFFIT | 0.1 IU/mL | Dogs with preexisting Ab: increase for 3/3, naive dogs: 14/23 became seropositive | Dogs naive: 11/23 Ab>0.5 |  |  |  |  | [28] |
|  | 14 | Feral dogs | Various status |  | Sweet flavored Ultralite bait |  |  |  | Dogs with preexisting Ab: increase for 3/5, naive dogs: 4/7 became seropositive | Naive dogs: 2/7 Ab >0.5 |  |  |  |  |  |
| CAV-2-E3Δ-RGP | 20 | 75-100 days old beagle dogs | Seronegative on D0 | China | Sub-cutaneous | 1 x 10^7.8^ TCID_50_  on D0 and D14 | FAVNT |  |  | 20/20 | 25 weeks | 20/20 | 6 months | 0/20 | [29] |
| CAV-2-E3Δ-RGP | 13 | 75-100 days old beagle dogs | Seronegative on D0 |  | Sub-cutaneous | 1 x 10^7.8^ TCID_50_ on D0 |  |  |  | 13/13 |  | 13/13 |  | 0/13 |  |
| CAV-2 (control) | 20 | 75-100 days old beagle dogs | Seronegative on D0 |  | Sub-cutaneous | 1 x 10^7.8^ TCID_50_ on D0 |  |  |  | 0/20 |  | 0/20 |  | 20/20 |  |
| CAV-2-E3Δ-RGP | 46 | Housekeeping indigenous dogs | Never vaccinated against rabies | China | Intra-nasal | 1 x 10^8.5^ PFU | FAVNT |  | 40/46 (87.0%) |  |  |  |  |  | [30] |
|  | 90 | Housekeeping indigenous dogs | Never vaccinated against rabies |  | Bait | 3 x 10^8.5^ PFU |  |  | 79/90 (87.8%) |  | 2 years | 10/10 | 15 weeks |  |  |
|  | 10 | Housekeeping indigenous dogs | Never vaccinated against rabies |  | Control | - |  |  | 0/10 |  |  | 1/10 | 15 weeks |  |  |
| SAD Bern | 7 | Indigenous tunisian dogs | Not previously vaccinated and seronegative on D0 | Tunisia | Oral instillation | 10^7.5^ TCID_50_ | RFFIT |  |  | 7/7 | 120 days | 7/7 | 45 days after the last death in the control group | 0/7 | [19] |
|  | 7 | Indigenous tunisian dogs | Not previously vaccinated and seronegative on D0 |  | Oral instillation | 10^6.5^ TCID_50_ |  |  |  | 6/7 | 120 days | 7/7 | 45 days after the last death in the control group | 0/7 |  |
|  | 4 | Indigenous tunisian dogs | Not previously vaccinated and seronegative on D0 |  | Control | - |  |  |  | 0/4 | 120 days | 0/4 |  | 4/4 |  |
| rERAG333E | 10 | Beagle aged 3 months | Seronegative on D0 | China | Oral instillation | 10^8.0^ PFU | FAVNT |  | 9/10 (W4), 7/9 (W52) |  |  |  |  |  | [31] |
|  | 10 | Beagle aged 3 months | Seronegative on D0 |  | Oral instillation | 10^9.0^ PFU |  |  | 10/10 (W4), 8/10 (W52) |  |  |  |  |  |  |
| SPBNGAS-GAS | 27 | 3-12 months indigenous dogs | Seronegative on D0 | Haiti | Bait | ND | RFFIT | 0.05 IU/mL | 16/27 (59.3%) |  |  |  |  |  | [32] |
| VRC-RZ2 | 8 | 3-12 months | Seronegative on D0 | Kazakhstan | Bait | 10^6.7^ TCID_50_ | MNT | 0.5 IU/mL | 8/8 | 8/8 |  |  |  |  | [33] |
|  | 4 | 3-12 months | Seronegative on D0 | Kazakhstan | Bait | 10^6.7^ TCID_50_ | MNT | 0.5 IU/mL | 4/4 | 4/4 | 180 days | 4/4 | 90 days |  |  |
|  | 2 | 3-12 months | Seronegative on D0 | Kazakhstan | Control | - | MNT | 0.5 IU/mL | 0/2 | 0/2 | 180 days | 0/2 | 90 days |  |  |
