# Supplementary material for: Oral vaccination of dogs: a well-studied and undervalued tool for achieving human and dog rabies elimination
Source: Vet Res. 2018 Jul 13;49:61. doi: 10.1186/s13567-018-0554-6 (PMC6045873; doi:10.1186/s13567-018-0554-6)
Supplement: Supplementary file 2 — Additional file 2. Attractiveness studies. This table contains compiled information summarizing attractiveness data on oral rabies vaccines for dogs studies. [file 13567_2018_554_MOESM2_ESM.docx]

Additional file 2: Attractiveness. DBL2: freeze-dried SAG2 bait, ND: Not Documented, PFU: Plaque Forming Units, DM: Sulfadimethoxine, TCID50: median Tissue Culture Infectious Doses, WIM: Wildlife Immunization Model

| **Vaccine** | **Nb dogs** | **Age** | **Country** | **Administration** | **Dose per animal** | **Delivery** | **Biomarker** | **Attractiveness** | **Source** |
| --- | --- | --- | --- | --- | --- | --- | --- | --- | --- |
| SAG2 | 7 | < 3 months (N=1), 3-12 months (N=1), > 1 year (N=5) | Tunisia | DBL2 bait | 10^8.3^ TCID_50_ | Fed in animal facility |  | 6/7: whole bait consumed, 1/7 partly consumed | [34] |
| SAG2 | 9 | 6-12 months | India | DBL2 bait | 10^8.5^ TCID_50_ | Fed in animal facility |  | 21/21: whole bait consumed (17 < 3 min,  4 < 20 min) | [24] |
| SAG2 | 5 | Laboratory adult beagles | CDC, US | Rabigen Oral | 10^8.2^ TCID_50_ | Fed in animal facility |  | All sachets chewed thoroughly or punctured | [23] |
|  | 3 | Laboratory adult beagles |  | Rabigen Oral | 10^7.5^ TCID_50_ | Fed in animal facility |  |  |  |
|  | 5 | Laboratory adult beagles |  | Rabigen Oral | 10^7.4^ TCID_50_ | Fed in animal facility |  |  |  |
|  | 5 | Laboratory adult beagles |  | DBL2 bait | 10^8.3^ TCID_50_ | Fed in animal facility |  | Baits completely eaten, except 1 partly eaten |  |
|  | 4 | Laboratory adult beagles |  | DBL2 bait | 10^7.2^ TCID_50_ | Fed in animal facility |  |  |  |
|  | 4 | Laboratory adult beagles |  | DBL2 bait | 10^6.9^ TCID_50_ | Fed in animal facility |  |  |  |
| - | 286 | Puppies, juveniles, adults (70%) | Tunisia (field study) | DBL2 bait | - | Mobile vaccination centre | SDM | 85-90% dogs in the study area: bait consumed at least partially | [46] |
| - | 335 | Indigenous dog > 2 months | Tunisia (field trial) | DBL2 bait | - | Door-to-door baiting | Rhodamine B | 59.1% dogs after campaign: took the bait and rhodamine B positive. 67.5% dogs to which a bait was presented: at least partial acceptance | [47] |
|  |  |  |  |  |  | Transect line baiting (WIM) | SDM | 58.7% baits disappeared within 24 hours, 24.1% owned dogs SDMpositive, > 40% free-roaming dogs positive consumed baits (ownerless and owned dogs): |  |
| SAG2 | 60 | Owned dogs | Marocco | Chicken-liver flavoured bait (placebo) | - | Door to door | Methylene-blue | 77% dogs: baits consumed (< 3 min), 62%: capsule pierced | [70] |
|  | 15 | Stray dogs |  | Chicken-liver flavoured bait (placebo) | - | Hand-out | Methylene-blue | 46.7% dogs: baits taken |  |
|  |  | Stray dogs |  | Chicken-liver flavoured bait + sachet | 10^7.8^ TCID_50_ | WIM on transect lines |  | up to 73% baits disappeared overnight, up to 68% capsules perforated |  |
| - | ? | Household dogs | Mexico | Dog biscuit, milk/egg/corn flavoured bait, beef sausage | - | Door-to-door |  | 71-96% baits eaten by household dogs | [53] |
|  | 20 | Confined dogs | CDC, US | Several baits with diffent flavours | - | CDC facility |  | 65-91% baits eaten by confined dogs |  |
|  | 30-40 | Farmer-owned dogs | Egypt | Several baits with diffent flavours | - | Hand-out |  | 32-88% baits eaten by farmer-owned dogs |  |
| V-RG | 1242 | > 3 months household dogs | Sri Lanka | Bait (fishmeal) | ND | Door to door |  | 80% dogs: bait at least partly consumed | [64] |
|  |  |  |  |  |  |  |  | 53% dogs: considered as vaccinated (bait chewed + sachet punctured) |  |
| V-RG | 9 | Beagles: 9-11 months | France | Rectangular bait | 10^8.4^ TCID_50_ | Fed in animal facility |  | 9/9:whole bait consumed [ < 1h (N=8), 24h (N=1) ] | [27] |
|  | 9 | Beagles: 9-11 months |  | Square bait | 10^8.4^ TCID_50_ | Fed in animal facility |  | 9/9:whole bait consumed [ < 1h (N=8), 24h (N=1) ] |  |
|  | 3 | Beagles: 9-11 months |  | Rectangular placebo bait | - | Fed in animal facility |  | 3/3:whole bait consumed [ < 1h (N=2), 24h (N=1) ] |  |
|  | 3 | Beagles: 9-11 months |  | Square placebo bait | - | Fed in animal facility |  | 3/3:whole bait consumed [ < 1h (N=1), 24h (N=2) ] |  |
| V-RG | 42 | Livestock guardian dogs belonging to packs | Israel | Fishmeal polymer bait | > 10^8.0^ TCID_50_ | Hand-out |  | 46% baits swallowed, 28% sachets punctured | [66] |
|  | 42 | Livestock guardian dogs belonging to packs |  | Coated sachet |  |  |  | 20% baits swallowed, 12% sachets punctured |  |
| SAG2 | 753 | Owned dogs | South Africa | DBL2-Rabidog bait | ND | Door to door |  | 76.8% dogs: baits at least partly consumed (in 2.4 min on average) | [68] |
| SAD B19 | 6 | 3-10 months free-roaming indigenous dogs | Turkey | Köfte-bait | 10^8.2^ PFU | Dog shelter |  | 5/6 bait consumed in less than 5 min, 1/6 in 2 hours | [21] |
|  | 6 |  |  | Intestine bait | 10^8.2^ PFU |  |  | 5/6 bait consumed in less than 5 min, 1/6 in 2 hours |  |
| - |  | > 3 months | Turkey | Several dog baits |  | Hand-out |  | 7.7% to 96.3%: baits at least partly consumed (Köfte-bait > softcheese bait > chickenleg köfte-bait > chicken head köfte-bait). | [60] |
| - | 26 | Feral dogs | Navaro Nation facility, U.S. | Different flavors Ultralite baits |  | Hand-out |  | Consumption: Bacon (84.6%), cheese (81.7%), fish (80.7%), egg (79.9%), and sweet (53.8%). | [28] |
| - | 741 | Feral dogs | Navaro and Hopi Nations, U.S. | Commercially available baits |  | Hand-out |  | Chewing and puncture of the sachet: 75.6% for the fish-meal-crumbled coated sachet, 58.0% for the fish-meal polymer (Bait-Tek), 53.3% for the dog food polymer (Bait-Tek) and only 37.9% for Ontario slim | [65] |
| - | 261 | Owned and feral dogs | Guatemala | Several baits + attractants |  | Hand-out |  | 50.1% to 87.1%: bait at least partly consumed | [59] |
| - | 13 | 6 months to 4 years, free-roaming dogs | Navajo Nation Animal Control facility | 7 bait flavors (bacon, cheese, dog food, hazelnut, sugar-vanilla, peanut butter, and sardine) added to a commercial bait matrix |  |  |  | Selection: dog food (56% of time), bacon (54%), cheese (49%), sardine (45%), peanut butter (29%), hazelnut (19%), sugar vanilla -14%) | [67] |
| - | 116 | >3 months owned free-roaming dogs | The Philippines | Baits made locally (chickenneck, intestine, boiled intestine) |  | Hand-feeding (house to house) |  | Consumption: boiled intestine (93%), raw intestine (72%), chickenneck (63%) | [61] |
|  |  |  |  |  |  |  |  | Bait accepted and capsule punctured: boiled intestine (87.3%), intestine (54.8%), chickenneck (53.5%) |  |
| SAD B19 | 126 | > 2 months owned dogs | The Philippines | Boiled intestine bait | 10^8.4^ PFU | Hand-out |  | Bait accepted by 96.1% of dogs, accepted and capsule punctured or chewed (90%) | [61] |
| - | 200 | Adult and juvenile indigenous household dogs | Tunisia | Chickenhead, fishmeal bait, sausage, sponge bait with attractants | - | Door to door | Rhodamine B | Bait accepted: 96% chicken head, 80% fishmeal, 66% sponge, 56% sausage | [56] |
|  |  |  |  |  |  |  | or methylene blue | Biomarker contact: 97.9% chickenhead, 77.5% fishmeal, 46.4% sausage, 30.3% sponge |  |
|  |  |  |  |  |  |  |  | Potential success of vaccination: 94% chickenhead, 62% fishmeal, 26% sausage, 20% sponge |  |
| - | Adult (83%), Juvenile (12%), Pupuy (5%) | Owned, stray or community dogs, restricted or free-roaming | Navaro Nation reservation | Egg-flavored bait, boiled bovine intestine, fishmeal bait |  | Hand-out |  | Bait acceptance: 91.9% for intestine bait, 81.1% for fishmeal, 77.4% for egg-flavored bait | [55] |
|  |  |  |  |  |  |  |  | Biomarker contact: 75.4% intestine baits, 68.0% egg-flavored bait, 54.3% fishmeal |  |
| SPBNGAS-GAS | 235 | 3-12 months indigenous dogs | Haiti | Bait |  | Hand-out | ND | Bait accepted : 80.8% for dogs on private poverty | [32] |
|  | 50 | 3-12 months indigenous dogs | Haiti | Bait |  | Hand-out |  | Bait accepted : 17.2% for dogs in the street |  |
